# The value of PROMs for predicting erectile dysfunction in prostate cancer patients with Bayesian network

**DOI:** 10.1016/j.tipsro.2024.100234

**Published:** 2024-02-13

**Authors:** Biche Osong, Hajar Hasannejadasl, Henk van der Poel, Ben Vanneste, Joep van Roermund, Katja Aben, Johan Van Soest, Inge Van Oort, Laura Hochstenbach, Esther J. Bloemen- van Gurp, Andre Dekker, Rianne R.R. Fijten

**Affiliations:** a Department of Radiation Oncology (MAASTRO), Department of Radiation Oncology (MAASTRO), GROW School for Oncology and Reproduction, Maastricht University Medical Centre, Maastricht, the Netherlands; b Department of Urology, Netherlands Cancer Institute, Amsterdam, the Netherlands; c Department of Urology, Maastricht University Medical Center, the Netherlands; d Department of Research Development, Netherlands Comprehensive Cancer Organization, Utrecht, the Netherlands; e Department of Urology, Radboud university Medical Center, Nijmegen, the Netherlands.

## Abstract

**Purpose:**

This study aims to develop and externally validate a clinically plausible Bayesian network structure to predict one-year erectile dysfunction in prostate cancer patients by combining expert knowledge with evidence from data using clinical and Patient-reported outcome measures (PROMs) data. In addition, compare and contrast structures that stem from PROM information and routine clinical data.

**Summary of background:**

For men with localized prostate cancer, choosing the optimal treatment can be challenging since each option comes with different side effects, such as erectile dysfunction, which negatively impacts their quality of life. Our previous findings demonstrate that logistic regression models are able to identify patients at high risk of erectile dysfunction. However, methods such as Bayesian networks may be more successful, as they intricately represent the causal relations between the variables.

**Patients and methods:**

946 prostate cancer patients from 65 Dutch hospitals were considered to develop the Bayesian network structure. Continuous variables were discretized before analysis based on expert opinions and literature. Patients with missing information and variables with more than 25% of missing information were excluded. Prostate cancer treating physicians first determined the relationships (arcs) between the available variables. The structures were then modified based on algorithmically derived structures using the hill-climbing algorithm. Structural Performance was evaluated based on the area under the curve (AUC) values and calibration plots on the training and test data.

**Results:**

BMI and prostate volume via MRI were excluded from this analysis due to their high percentage of missingness (*>*45 %). The final cohort was reduced to 505 and 216 after excluding 157 and 68 patients with missing information, respectively. The AUC of the PROM structure was better than the clinical structure in both the train and test data. The structure that combined both sources of information had an AUC value of 0.94 (0.92 – 0.96) and 0.84171 (0.77 91) in the train and test data, respectively.

**Conclusion:**

Bayesian network structures derived from PROM information by complimenting expert knowledge with evidence from the data produce a clinically plausible structure that is more performant than structures from clinical data. Our study supports the growing global recognition of incorporating the patient’s perspective in outcomes research for better decision-making and optimal outcomes. However, a structure that combines both sources of information gives a more holistic view of the patient with actionable insights and improved discriminative power.

## Highlights

• PROMs are more predictive than clinical information for endpoints like erectile dysfunction.
• Patient perspective should be incorporated in outcome research, such as erectile dysfunction, since the patient understands the disease symptoms better.
• Experts' involvement in clinical research is critical as it aligns the modeling steps with the clinical process for increased clinical plausibility.

## Introduction

Erectile dysfunction, also called impotence, is the inability to develop or sustain an erection satisfactory for sexual intercourse [1]. It is one of the most common types of sexual dysfunction and affects approximately 50 % of men above the age of 40 years and increases to about 70 % for men 70 years and older. Erectile dysfunction is primarily linked with prostate cancer and the psychiatric disorders caused by disease diagnosis [2], [3]. However, a large majority of erectile dysfunction is caused by the numerous treatment options of prostate cancer such as surgery (prostatectomy), radiation, and hormonal therapy because the nerves and blood vessels that control the physical aspect of an erection are incredibly delicate and easily affected by any change or trauma to the area [2].

Whether or not the nerves were spared during surgery or whether the most precise dose planning was used during radiation therapy, erectile dysfunction remains the most common side effect of prostate cancer treatment and the predominant concern for a significant majority of patients with localized prostate cancer, since it negatively impacts their quality of life [3]. Therefore, knowing if a prostate cancer patient will have erectile dysfunction after treatment is pivotal for shared decision-making at the same time, choosing the optimal treatment can be challenging since each option comes with different side effects, erectile dysfunction included [4].

In recent years, caregivers have been making use of patient-reported outcome measures (PROMs) to evaluate patients’ health and well-being during the treatment process [5], [6]. These PROMs capture patients’ information such as symptoms, performance status, and quality of life which is based on the patient’s perspective of their health at intervals [7]. Caregivers can then leverage this information to improve shared decision-making and better patient-centered care [8]. However, the amount of information available is more than humans can process, mostly because humans’ ability to handle several cognitive entities at a time is very limited [9]. This calls for support from predictive models that can better combine several important patient information to probabilistically determine the risk of them developing erectile dysfunction after treatment.

Verma *et al.* [10] have demonstrated that various models can be successfully applied to PROM information to predict short- and long-term outcomes to guide shared decision-making processes between clinicians and patients for better cancer care. In addition, Rivera *et al.* [11] have also stressed the importance of integrating PROM in healthcare outcome research studies since it allows for the incorporation of the patient’s view of the disease symptoms, hence reflecting a more holistic picture of the patient’s health and well-being for optimal decision. Our previous findings demonstrate that logistic regression can nicely combine PROM and clinical information to identify patients liable for developing erectile dysfunction [12]. However, only two PROMs were included in the model, which does not provide the full predictive potential of PROM information. Moreover, no study has leveraged the power of algorithms such as Bayesian networks on PROM information, which can intricately represent the causal relations between the available variables and efficiently make probabilistic inferences about any variable of interest in the network with optimal performance [13], [14]. In addition, they have increased clinical plausibility if specified by experts in the field and are more performant when inferred from the available data (evidence from data) by a learning algorithm [13].

Therefore, this study aims to develop and externally validate a performant and clinically plausible Bayesian network structure to predict one-year erectile dysfunction in prostate cancer patients by complementing expert knowledge with evidence from data (expert-modified structure).

We hypothesize that though a Bayesian network can offer a more holistic view of a prostate cancer patient’s treatment journey because it can incorporate more patient information and the interplay between these variables, a carefully developed Bayesian network structure using PROM information will outperform a structure developed from clinical information.

## Materials and methods

This study analyses a subset of the ProZIB dataset collected by the Netherlands Comprehensive Cancer Organization (Integraal Kankercentrum Nederland; IKNL) from 65 Dutch hospitals. More information about the complete ProZIB dataset is described in the article by Vernooij *et al.* [15]. This subset contained clinical information of 964 localized prostate cancer patients with their PROMs information based on the EPIC26 questionnaire (Supplementary materials) at diagnosis, 1 and 2 years after diagnosis. Only patients with localized or locally advanced prostate cancer were included in this study. Patients with regional lymph node involvement or distant metastasis (cT1-3, N0, M0) were excluded. Also, we excluded all patients with missing information and variables with more than 25 % missingness. All the patients in this study provided written informed consent to participate.

Patient information from each hospital was randomly assigned to the training or test data to develop the Bayesian network structures and test their performance, respectively. Table 1, Table 2 show the PROMS and clinical variables under consideration. Erectile dysfunction at one year was considered the endpoint of interest, dichotomized in patients with no sexual problems and patients with any sexual problems as shown in equation 1. The Sankey diagram (Fig. S1 supplementary materials) shows the flow changes in patients’ frequency of erections at baseline and 1-year.

**Figure:**
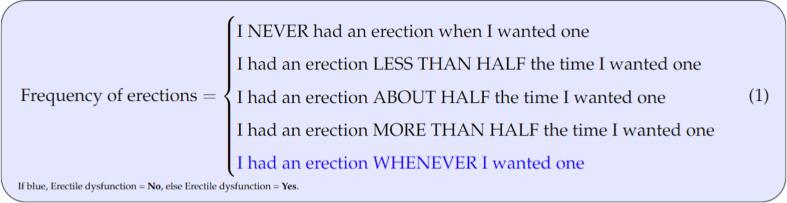

**Table 1:** General PROMs characteristics at baseline on the training and validation datasets with the dichotomized outcome of interest at 1-year.

Variable	Levels	Training		Validation		p-value
Ability to have erection	Very poor		156 (23.6 %)	63 (22.2 %)		0.99
	Poor		69 (10.4 %)	29 (10.2 %)		
	Fair		148 (22.3 %)	67 (23.6 %)		
	Good		227 (34.3 %)	97 (34.1 %)		
	Very good		44 (6.7 %)	21 (7.4 %)		
	*Missing*		18 (2.7 %)	7 (2.5 %)		
Ability to reach orgasm/climax	Very poor		175 (26.5 %)	55 (19.4 %)		0.12
	Poor		65 (9.8 %)	32 (11.3 %)		
	Fair		120 (18.1 %)	69 (24.3 %)		
	Good		244 (36.9 %)	101 (35.6 %)		
	Very good		38 (5.7 %)	17 (5.9 %)		
	*Missing*		20 (3.0 %)	10 (3.5 %)		
Quality of erections	None at all		99 (14.9 %)	32 (11.3 %)		0.61
	Not firm enough^∗^		88 (13.3 %)	38 (13.4 %)		
	Firm enough^†^		162 (24.5 %)	78 (27.5 %)		
	Firm enough^††^		294 (44.4 %)	128 (45.1 %)		
	*Missing*		19 (2.9 %)	8 (2.8 %)		
Frequency of erections	Never when I wanted one		128 (19.3 %)	52 (18.3 %)		0.88
	*<*50 % when I wanted one		80 (12.1 %)	37 (13.0 %)		
	50 % when I wanted one		62 (9.4 %)	33 (11.6 %)		
	*>*50 % when I wanted one		103 (15.6 %)	40 (14.1 %)		
	Whenever I wanted one		260 (39.3 %)	108 (38.0 %)		
	*Missing*		29 (4.4 %)	14 (4.9 %)		
Ability to function sexually	Very poor		81 (12.2 %)	37 (13.0 %)		0.24
	Poor		143 (21.6 %)	46 (16.2 %)		
	Fair		191 (28.9 %)	101 (35.6 %)		
	Good		193 (29.2 %)	77 (27.1 %)		
	Very good		31 (4.7 %)	11 (3.9 %)		
	*Missing*		23 (3.5 %)	11 (4.2 %)		
Sexual functioning (How big a problem)	No problem		335 (50.6 %)	133 (46.8 %)		0.83
	Very small problem		113 (17.1 %)	58 (20.4 %)		
	Small problem		135 (20.4 %)	58 (20.4 %)		
	Moderate problem		50 (7.6 %)	21 (7.4 %)		
	Big problem		9 (1.4 %)	4 (1.4 %)		
	*Missing*		20 (3.0 %)	10 (3.5 %)		
Feeling depressed	No problem		523 (79.0 %)	242 (85.2 %)		0.15
	Very small problem		72 (10.9 %)	22 (7.8 %)		
	Small problem		30 4.5 %)	6 (2.1 %)		
	Moderate problem		12 (1.8 %)	2 (0.7 %)		
	Big problem		3 (0.5 %)	2 (0.7 %)		
	*Missing*		22 (3.3 %)	10 (3.5 %)		
Erectile dysfunction at 1-year	No		140 (21.2 %)	54 (19.0 %)		0.45
	Yes		486 (73.4 %)	215 (75.7 %)		
	*Missing*	6	36 (5.4 %)	15 (5.3 %)		

∗: For sexual activity, †: For masturbation/foreplay, ††: For intercourse.

**Table 2:** General patient clinical characteristics at baseline on the training and validation datasets with the dichotomized outcome of interest at 1-year.

**Variable**	**Levels**		**Training**	**Validation**		**p-value**
Patient age	Median (sd)		69 (6.6)	69 (7)		0.85
PSA^∗∗^ level at diagnosis	Median (sd)		7.9 (13.0)	8.1 (15.2)		0.39
	*Missing*		9 (1.4 %)	5(1.8 %)		
Volume of prostate^#^	Median (sd)		48 (24.8)	54 (27)		0.19
	*Missing*		505 (76.3 %)	218 (76.8 %)		
Body mass index (BMI)	Median (sd)		26.4 (2.7)	26.7 (3.4)		0.19
	*Missing*		327 (49.4 %)	157 (55.3 %)		
Tumor N stage	N0		371 (56.0 %)	171 (60.2 %)		0.24
	NX^∗^		291 (43.9 %)	113 (39.8 %)		
Tumor T stage	T1		314 (47.4 %)	120 (42.2 %)		0.12
	T2		267 (40.3 %)	116 (40.9 %)		
	T3		81 (12.3 %)	48 (16.9 %)		
Charlson Comorbidity Index (CCI)	None		416 (62.8 %)	191 (67.3 %)		0.56
	1 point		137 (20.7 %)	53 (18.7 %)		
	≥2 points		99 (14.9 %)	35 (12.3 %)		
	*Missing*		10 (1.5 %)	5 (1.7 %)		
Gleason group	Group 1		379 (57.3 %)	155 (54.6 %)		0.34
	Group 2		160 (24.2 %)	66 (23.2 %)		
	Group 3		56 (8.5 %)	24 (8.5 %)		
	Group 4		48 (7.2 %)	23 (8.1 %)		
	Group 5		19 (2.8 %)	16 (5.6 %)		
Diabetes	No		587 (88.7 %)	250 (88.1 %)		0.78
	Yes		75 (11.3 %)	34 (11.9 %)		
Cardiovascular disease	No		313 (47.3 %)	139 (48.9 %)		0.67
	Yes		349 (52.7 %)	145 (51.1 %)		
Hormone therapy	No		614 (92.7 %)	256 (90.1 %)		0.18
	Yes		48 (7.3 %)	28 (9.9 %)		
Alcohol use	No		66 (9.9 %)	30 (10.5 %)		0.78
	Former		41 (6.2 %)	13 (4.6 %)		
	Yes		477 (72.1 %)	209 (73.6 %)		
	*Missing*		78 (11.8 %)	32 (11.3 %)		
Smoking status	Non smoker		281 (42.5 %)	125 (44.0 %)		0.95
	Former smoker		259 (39.1 %)	108 (38.0 %)		
	Current smoker		42 (6.3 %)	19 (6.7 %)		
	*Missing*		80 (12.1 %)	32 (11.3 %)		
Therapy	Brachytherapy		39 (5.9 %)	20 (7.0 %)		0.59
	EBRT		137 (20.7 %)	67 (23.6 %)		
	No Therapy		277 (41.8 %)	108 (38.0 %)		
	Prostatectomy		209 (31.6 %)	89 (31.3 %)		
Erectile dysfunction at 1-year	No		140 (21.2 %)	54 (19.0 %)		0.45
	Yes		486 (73.4 %)	215 (75.7 %)		
	*Missing*		36 (5.4 %)^7^	15 (5.3 %)		

∗∗: prostate-specific antigen, #: Via MRI, ∗: Unknown.

### Bayesian network

Bayesian networks are graphical models called directed acyclic graphs (DAG) where the available variables of a system, domain, or organ under consideration are represented as nodes, and directed arrows represent probabilistic dependencies or causal relationships between the variables in a graphical [16], [17]. In a DAG, if an arrow or arc moves from node **X** to node **Y**, then node **X** has an influence on node **Y**. Hence, Nodes **X** is considered to be the parent of node **Y**, and node **Y** is said to be the child of nodes **X**. A variable can be a parent or child to several variables but can not act as a parent and child at the same time to another variable. The quantitative connection between parents and children in a DAG is provided by the conditional probability table (CPT), which shows the probabilistic state of a node based on the states of its parent node(s) [16], [18].

### Statistical analysis

Exploratory analyses and data visualization were applied to understand the data and detect any underlying patterns necessary to construct the Bayesian network, such as missing information and possible outlying values. All continuous variables were categorized either based on experts’ opinions or from literature. All patients with missing information and variables with missingness above 25 % were excluded from the analysis. From the 65 Dutch hospitals, we randomly reserved data from.

45 hospitals (≈ 70 %) to train the CPT of the resulting Bayesian network structures and 20 hospitals (≈ 30 %) to test their performance. The predictive performance of the Bayesian network structures was assessed by computing the area under the curve (AUC) and generating calibration plots. All analyses were conducted in R version 4.2.1 [19] with the bnlearn package [20], [21] to develop the Bayesian network structures and the Graphical Network Interface (GeNIe) application visualization [22].

### Structure learning

Expert prostate cancer treating physicians specified the relationship between the variables in the Bayesian network based on the PROM and clinical information separately. Separate structures were also inferred from form each of the data sources using the hill-climbing (HC) structure learning algorithm and Bayesian Information Criterion for the goodness-of-fit measure. These algorithmic structures and literature searches were used to complement the expert-specified structures by either adding, removing, or reversing arcs in the expert structure (expert-modified structure). Also, variables without a link to the outcome based on the algorithmic structure were removed from the expert structure. The final structure is a combination of the expert-modified structures based on the algorithmic structures from both data sources. The performance of the resulting Bayesian network structures was evaluated by computing the area under the curve (AUC) and generating calibration plots.

### Structure comparison

To test our hypothesis, we compared the performance of the expert-modified Bayesian network structures in predicting erectile dysfunction at one year for prostate cancer patients. To make the comparison fair, we used the same learning algorithm and goodness-of-fit measure to develop the algorithmic structure for both data sources, which are used to complement the expert structures. The final expert complimented structures were first compared structural appearance and then based on performance measures such as the AUC, sensitivity, and specificity values. Calibration plots were generated to compare which structure had its predicted probabilities closer to the observed frequency in data.

## Results

Based on the data from the different Dutch hospitals, a total of 946 prostate cancer patients were considered for this study and grouped as follows. Data from the randomly selected 45 hospitals resulted in a total of 662 patients, which were assigned to train the structures, and the rest 20 hospitals with 284 patients to test the performance. From the train and test data, 157 and 68 patients with missing information were excluded from further analysis, reducing the cohort to 505 and 216, respectively. There was no statistical difference between patients in the training and test cohorts for all the considered variables. The BMI and volume of prostate via MRI variables were excluded from further analysis due to their high percentage of missing information.

The median age of patients in this study was 69 (45–––87). Most of the patients in this study are nonsmokers or ex-smokers, with more than half of the patient population being alcohol users. Most of the patients in this study either received surgery or no therapy (watch and wait) with only a handful administered hormonal therapy. In general, most of the patients seem to be in ”good health” since a majority of them had no CCI, diabetes, depression, or sexual problems. However, about 50 % of the patients had a cardiac problem. The outcome of interest was unbalanced, with 73 % and 75 % of the patients experiencing erectile dysfunction in the train and test data, respectively. The median prostate-specific antigen was 7.9, with a standard deviation of 13 in the training dataset. For BMI, the median value was 26.4 with a standard deviation of 2.7 which falls in the overweight category. The median value of the volume of prostate, on the other hand, was almost twice (48) the normal prostate volume on scan with a standard deviation of 24.8. Table 1, Table 2 show a detailed description of patients’ characteristics for this study cohort.

There was a significant mean difference (p-value *<* 0.05) between patients with and without erectile dysfunction for age and prostate-specific antigen variable (Fig. S3 supplementary materials). The age variable was categorized into two groups with a cutoff value of 70 years. The prostate-specific antigen (PSA) was discretized into three groups based on experts’ suggestions with cutoff values at 4 ng/ml and 10 ng/ml (Fig. S1 supplementary materials). The number of patients without erectile dysfunction was higher in the”good sex life” groups (Patients who responded either, good, no problem, firm enough, or whenever to the questionnaire items) compared to patients who responded otherwise (Fig. S4 supplementary materials). However, the number of patients in the”good sex life” groups with erectile dysfunction was equally very high. A similar trend is also observed with the clinical variables as most of the patients without erectile dysfunction have a relatively better health condition or lifestyle except for the alcohol use variable, where most of the patients were in the drinking group and cardiovascular disease, which had almost the same number of patients (Fig. S5 supplementary materials). Based on treatment, most of the patients without erectile dysfunction did not receive any active therapy.

The experts specified clinical structure had all but four (PSA, Tstage, Nstage, and Gleason) variables being direct parents to the outcome. The algorithmic structure, on the other hand, the algorithmic DAG had two distinct structures, with only therapy being a parent to the outcome. Smoking status, alcohol use, diabetic status, CCI, and cardiovascular disease had no link to the outcome and were removed from the clinical expert-modified structure (Fig. S6 supplementary materials). The two structures were then compared to build the expert-modified structure by either removing (Therapy to Hormone therapy), maintaining (Gleason to therapy and therapy to outcome), reversing (PSA to hormone therapy and Age to Therapy), or adding (Tstage to Nstage and Age to PSA) certain arcs. The same procedure was followed to establish the PROM expert-modified (Fig. S7 supplementary materials) structure.

### Structure comparison

**Fig. 1:**
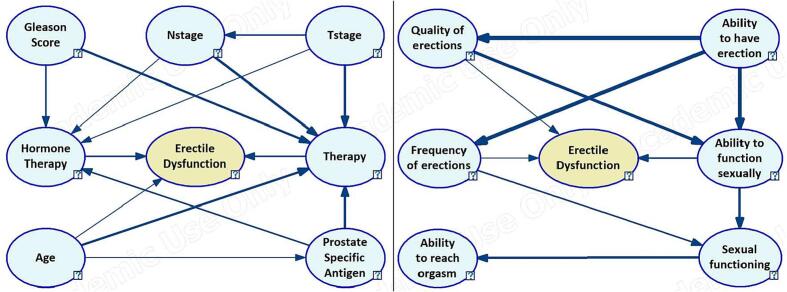
Expert-modified Bayesian network structures using clinical and PROM, respectively. The oval structure represents variables or Node, and arrows show the direction of the causal-effect relationships. The arrow size indicates the magnitude of influence.

Fig. 1 shows the resulting expert-modified Bayesian network structures. The clinical structure has 8 variables and 14 arcs, with the outcome having three parents. The PROM structure, on the other hand, has one variable less than the clinical structure with 10 arcs and three to the outcome. In the clinical structure, three variables (Therapy, Hormone therapy, and PSA) had 3 or more parents. In the PROM structure, only the outcome was a child to 3 variables. In both structures, Tstage and Ability to have erection were the only variables to parent more than 2 variables. Ability to reach orgasm was the only variable without a child besides the outcome. Ability to have erection was the most influential variable in the PROM structure, especially to Quality of erections and Frequency of erection. Quality of erections and Ability to have erection both highly influenced Ability to function sexually. On the other hand, all parents of therapy had a high influence, while only Gleason score and PSA had a high influence on Hormone therapy. Tstage had more influence on Nstage than on Hormone therapy.

Table 3 shows the numerical comparison of the expert-modified Bayesian network structures based on their performance in terms of the AUC (Fig. S8 supplemental material), sensitivity, and specificity values for the different information sources in the training and test data. The PROM structure performed better than the expert structure for all matrices of interest, especially in the training data. However, both structures had the same accuracy and specificity value in the test data.

**Table 3:** The Accuracy, AUC, sensitivity, and specificity values of the expert-modified structure for the clinical and PROM data source on the train and test data.

**Data**	**Source**	**Accuracy**	**AUC (95 % CI)**	**Sensitivity**	**Specificity**
**Train**	PROM	0.76	0.82 (0.79–––0.86)	0.77	0.76
	Clinical	0.70	0.76 (0.72–––0.81)	0.72	0.70
**Test**	PROM	0.75	0.82 (0.76–––0.88)	0.82	0.73
	Clinical	0.75	0.71 (0.63–––0.79)	0.81	0.73

**CI:** confidence interval.

**Fig. 2:**
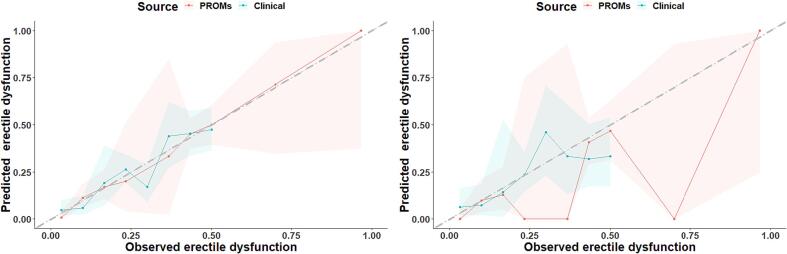
Calibration plots of the expert-modified Bayesian network structures in the training (left) and Test (right) data. The gray line represents ideal calibration, while the solid lines show the structure’s performance, and the colored bandwidths indicate their respective 95% confidence interval.

Fig. 2 shows the calibration plots of the expert-modified structures. Both structures seem to be well-calibrated in the train data. In the test data, the clinical structure looks to be better calibrated than the PROM structures since its predicted probabilities are more closely matched to the observed data frequencies. However, the clinical structure could not evaluate patients with higher probabilities but the PROM structure which could have a wider confidence interval.

**Fig. 3:**
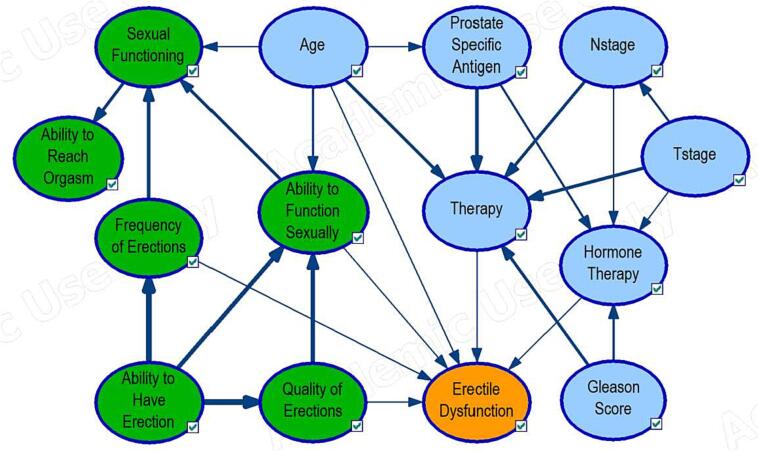
Bayesian network structures using clinical (Blue) and PROM (green) information. The oval structure represents variables or Node, and arrows show the direction of the causal-effect relationships. The arrow size indicates the magnitude of influence. (For interpretation of the references to colour in this figure legend, the reader is referred to the web version of this article.)

The final structure after combining the PROM and clinical expert-modified structures is shown in Fig. 3 with the addition of two arcs (Age to Ability to function sexually and sexual functioning). Therefore, the final structure comprises 14 variables and 26 arcs with six to the outcome. Only age was a parent to four variables.

**Fig. 4:**
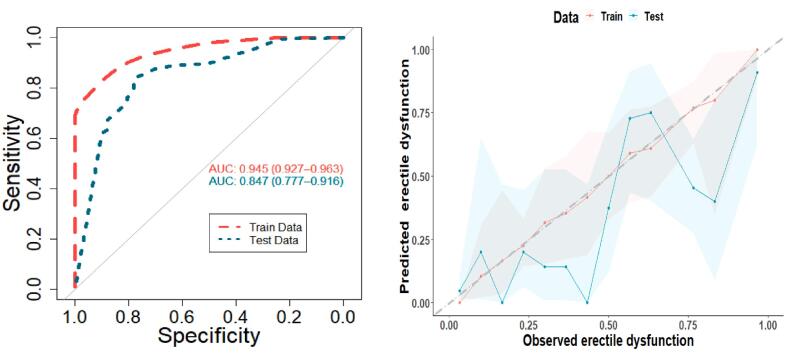
Area Under the Curve (AUC) and Calibration plots of the expert-modified Bayesian network structures, respectively. The gray line represents ideal calibration, while the solid colored lines show the structure’s performance on the training (red) and Test (cyan) data. The colored bandwidths on the calibration plot indicate the 95% confidence interval of the structures’ respective performance on the train and test data. (For interpretation of the references to colour in this figure legend, the reader is referred to the web version of this article.)

Fig. 4 shows the performance of the final structure in terms of AUC and calibration in the train and test data. The structure achieved an AUC value of 0.94 (0.92 – 0.96) and 0.84 (0.77–––91) for predicting the risk of erectile dysfunction at 1 year on the train and test data. The sensitivity and specificity values of the structure were 0.89 and 0.84, respectively, with an accuracy of 0.85 on the train data. In the test data, the sensitivity was 0.8 and the specificity 0.74, with an accuracy of 0.75. The calibration plots in Fig. 4 show the structure is well calibrated on the train data since predicted probabilities and the observed frequencies coincide almost at all points. On the test data, the structure seems to be poorly calibrated, given how distant the points are from the diagonal dotted gray line, which is the reference line for a perfectly calibrated structure.

## Discussion

In the current study, we have developed and validated a Bayesian network structure that uses both clinical and PROM information to predict erectile dysfunction for prostate cancer patients. The structure was developed to be clinically sane and sparse at the same time by combining knowledge from expert prostate cancer treating physicians and evidence from data. That is, including only variables with a direct or indirect relationship with the outcome of interest based on the algorithmically generated structure. The developed structure used seven clinical features and five PROM information known to have an influence on the sexual life of a patient. The predictive performance of the structure based on the AUC values was above 80 % in both the training and test data. However, the structure was not well-calibrated in the test data as compared to the training data due to the imbalanced nature of the data. The PROM expert-modified structure with one variable and four arcs less was more performant than the clinical expert-modified structure for all considered matrices in both the train and test data. However, both structures had the same accuracy and specificity in the test data.

Deist *et al.* [23] have shown that the choice of a model should not be based on performance since all models can capture the basic signal within any data with little difference in performance (AUC value) when properly analyzed and compared. These findings have also been recently confirmed by Hasannejadasl *et al.* [24] and Biecek *et al.* [25] on a limited number of models. However, these studies did not include Bayesian networks in their model comparison. Although Jayasurya *et al.* [26] and Ducher *et al.* [27] have shown that Bayesian networks have a superior predictive performance on SVM and logistic regression to predict IgA nephropathy and two-year survival in lung cancer patients, respectively. Bayesian network structure was used in this study due to the complexity of erectile dysfunction, which can be caused by hormones, emotions, nerves, muscles, blood circulation, stress, mental health, or a combination of these factors. Therefore, graphical models such as Bayesian networks with unlimited restrictions in defining interactions between variables are more suited to model such systems as they can better capture the biological process and complexity within the system. In addition, they can be built by experts who better understand the biological process behind the disease for more biological/clinical plausibility because two variables can be correlated but not necessarily have a causal effect. Also, their ability to make inferences about any variable in the network is very pivotal for this project, as the developed structure can support decision-making as both a diagnostic and prognostic tool. Lastly, no study has investigated the predictive value of Bayesian networks for erectile dysfunction in prostate cancer patients thus far.

The process involved in getting an erection is very complex, which, if disrupted at any step, can lead to erectile dysfunction problems. This complexity makes it challenging to predict if a patient will suffer from erectile dysfunction after treatment. Nonetheless, Hasannejadasl *et al.* [12] have also looked at predicting erectile dysfunction using logistic regression but a direct comparison with this study will not be fair because of the difference in the study design and analysis process. However, both studies do agree that the Frequency of erections, Quality of erections, Therapy, and Hormone therapy have a direct influence on erectile dysfunction. Gleason group and Tumor Tstage were also included as having a direct relationship with erectile dysfunction, which in this study, are only related via the type of treatment received by the patient. Alcohol use, lack of energy, Cardiovascular disease, and Diabetes were also included in their model, which was not in our final expert-modified structure. This difference in selected variables can be directly attributed to the model type and data preparation steps, given that they considered missing information as a level for some variables, which increases the sample size and the chance of getting a significant relationship with the outcome.

The social variables smoking status and alcohol use were not included in the final network despite known evidence to have an effect on erectile dysfunction. Smoking status is known to significantly increase the risk of erectile dysfunction given that it damages endothelial cells and impairs endothelial vasodilation [28], [29], [30], [31], [32]. However, their absence in the network can be understood because only about 6 % of the patients in this study are smokers. In addition, erectile function has been shown to greatly improve with smoking cessation, which accounts for about 39 % of the patient population in this study [32]. Alcohol consumption, on the other hand, is known to have an effect on erectile dysfunction because it damages blood vessels and nerves [33] and with more than 70 % of the patients in this study being alcohol consumers, its absence in the network is somewhat surprising. However, one might justify its absence by the fact that alcohol only affects erectile function when taken in excess, and this information was not available in the data. On the contrary, it temporarily affects hormone levels and increases sex drive with moderate consumption, which might explain in part the “good sex life” of most of the patients.

Other variables like the Charlson comorbidity index, cardiovascular disease, diabetes, and feeling depressed were also not included in the final structure, which is understandable because more than 70 % of the patients did not suffer from diabetes, feel depressed, or had any comorbidity. Although one might argue that the small percentage of depressed patients is due to antidepressants, this information was not captured in this study, and the relatively “good sexual life” of the patients proves otherwise since antidepressant medication should have a negative impact on users such as sexual dysfunction [34]. On the contrary, cardiovascular disease, which had about 50 % of the patients in the diseased group, was also not included in the final structure. Generally, the absence of these variables in the final structure can be attributed to the chosen goodness-of-fit measure for the learning algorithm, as it tends to prioritize sparse structures. However, Martell *et al.* [35] did not also find these excluded variables important to predict erectile dysfunction in a cohort of 212 patients receiving moderately hypofractionated radiotherapy to the prostate.

Most of the connections between the variables in the algorithmic structures (Supplementary materials Figs. S6 & S7) are either supported in the literature such as smoking status to alcohol use [36], Frequency of erections to erectile dysfunction [12], Therapy to erectile dysfunction [12], and diabetes to smoking status [37] or are clinically sane as Ability to function sexually is connected to Sexual functioning and Charlson Comorbidity Index is only linked to cardiovascular disease and Diabetes. This suggests that the HC algorithm combines well with the BIC scoring criteria as it penalizes structures with more parameters to avoid overfitting leading to a parsimonious structure that captures the signals (correlations) within the data better and hence can be used to complement the experts’ structure. However, this parsimony caused a well-established variable (feeling depressed) known to affect erection, not to be connected to erectile dysfunction directly or indirectly. Generally, depression is expected to lead to erectile dysfunction because of the complex sex-related chemistry between the brain and the sex organs, which is greatly disrupted in depressed persons [38].

Based on the clinical structure, all the disconnected variables share similar risk factors that involve blood flow obstruction. Normally, these variables should be associated with erectile dysfunction since they all share the same arterial cause [39], [40]. One might argue that the separation is due to the opted scoring criteria, which penalize complex models more the simple ones. However, these variables were still not connected to erectile dysfunction even with more lenient scoring criteria like the Akaike information criterion (AIC). One plausible explanation for this disconnection could therefore be that an important variable, such as endothelial dysfunction not considered in this study, could be the link between these disconnected variables and erectile dysfunction. Another reason could be that these variables are not directly related to erectile dysfunction but their interplay with other hematological variables not captured in this study increases their influence on erectile dysfunction [39], [40].

While it is important to develop accurate and performant models, it is also crucial that these models be sane, trustworthy, and interpretable, especially in a healthcare-related milieu. Therefore, using methods like Bayesian networks that can be developed by domain experts who understand better the biological process involved in the treatment of tumors and the aftermath would ensure that they are clinically sane [13] and their graphical nature facilitates their explanation to end users [41]. The objective of this study was therefore attended as the proposed Bayesian network structure, which combines PROMs and clinical information, has a predictive performance well above the chance level with a clinically plausible structure that can be easily understood and interpreted by clinicians given them the freedom to focus on addressing the medical concerns of the patient rather than struggling with comprehension of the structure. Therefore our proposed structure can serve as a decision aid to support personalized treatment decision-making in routine clinical settings. Our results also show that PROM information is better than clinical information for predicting erectile dysfunction in prostate cancer patients. This confirms our hypothesis and further supports the need to include patients’ perspectives in outcome research.

Our study is the first to assess the predictive value of Bayesian network structures for erectile dysfunction in prostate cancer patients to the best of our knowledge. Therefore, despite the performance of the Bayesian network structures, there is still room for improvement, especially in defining the relationship between the available variables. In addition, the performance was not optimal probability because we used disease-specific PROM information, which could restrict the structure’s performance since it will not capture all the vital functional well-being and health status of the patient. Hasannejadasl *et al.* [12] used lack of energy in their nomogram to predict erectile dysfunction, which shows to influence the outcome greatly. Also, the expert structures were modified with algorithmic structures developed on a collection of retrospective clinical data, which might be biased. Therefore, proper testing and validation of the structures are warranted before clinical applicability can be considered, preferably prospectively, on routine clinical data.

The retrospective nature of this study caused possible important variables such as BMI and volume of prostate via MRI not to be considered because of the high percentage of missing information. Skrypnik *et al.* [42] have shown that erectile dysfunction has a direct relationship with BMI, and patients with a high BMI are at increased risk of developing erectile dysfunction. The volume of prostate via MRI, on the other hand, is believed to be related to erectile dysfunction because an enlarged prostate causes several urinary problems, which can easily be extended to erectile dysfunction. However, we did not find any study that has investigated or found a direct relationship between an enlarged prostate and erectile dysfunction. Therefore an enlarged prostate by itself may not be the cause of erectile dysfunction, but rather the treatment for the enlarged prostate or other factors associated with it, like age as the prostate continues to grow with age [43]. Nonetheless, Collins *et al.* [43] found that prostate-specific antigen level is greatly influenced by the volume of the prostate. Such a link could have improved the performance of our final Bayesian network structure but was not included due to the retrospective nature of this study.

Given the pivotal role of blood in the process of erection, we believe that some hematological parameters such as Neutrophil-to-lymphocyte ratio, white-blood-cell count, lymphocyte count, and neutrophilic cell count, which are absent in the current study, could further improve the predictive performance of our Bayesian network [44]. Lastly, the outcome variable was dichotomized which leads to a loss of information. Future studies should consider using the different levels of erectile dysfunction to help patients and caregivers make optimal treatment decisions.

## Conclusion

We have developed and validated clinically plausible Bayesian network structures from routine clinical data, PROMs, and a combination of both data sources for predicting one-year erectile dysfunction in prostate cancer patients. The developed structures maximize performance and clinical plausibility as they complement experts’ opinions with evidence from data. Our result showed that PROM information is pivotal and better at predicting erectile dysfunction at one-year for prostate cancer patients, which further accentuates the growing global desideratum of incorporating patients’ perspectives in healthcare for quality and effective decision-making. However, combining both sources of information gives rise to a structure with improved discriminating power. In the future, we intend to update the developed Bayesian network structure with hematological parameters and then extend it to a dynamic structure to incorporate PROM information collected at different time points during treatment as this captures a more holistic view of the patient’s journey.

**Data availability statement**

Data used in this study were made available under contact between the different institutes and groups and the University of Sacred Heart, Rome (Italy). The agreements between the European and US institutions were based on the EU General Data Protection Regulation. Requests for datasets should be made to the original investigators from each cohort or trial with the pooled analysis.

## CRediT authorship contribution statement

**Biche Osong:** Conceptualization, Formal analysis, Investigation, Methodology, Visualization, Writing – original draft, Writing – review & editing. **Hajar Hasannejadasl:** Methodology, Visualization, Writing – original draft, Writing – review & editing. **Henk van der Poel:** Data curation, Funding acquisition, Project administration, Validation, Visualization, Writing – review & editing. **Ben Vanneste:** Data curation, Conceptualization, Funding acquisition, Investigation, Methodology, Resources, Supervision, Validation, Visualization, Writing – original draft, Writing – review & editing. **Joep van Roermund:** Data curation, Funding acquisition, Validation, Visualization, Writing – review & editing. **Katja Aben:** Data curation, Funding acquisition, Project administration, Validation, Visualization, Writing – review & editing. **Johan Van Soest:** Methodology, Resources, Supervision, Validation, Visualization, Writing – original draft, Writing – review & editing. **Inge Van Oort:** Data curation, Validation, Visualization, Writing – review & editing. **Laura Hochstenbach:** Project administration, Validation, Visualization, Writing – review & editing. **Esther J. Bloemen- van Gurp:** Funding acquisition, Project administration, Validation, Visualization, Writing – review & editing. **Andre Dekker:** Methodology, Resources, Resources, Supervision, Validation, Visualization, Writing – original draft, Writing – review & editing. **Rianne R.R. Fijten:** Funding acquisition, Methodology, Project administration, Resources, Supervision, Validation, Visualization, Writing – original draft, Writing – review & editing.

## Declaration of Competing Interest

The authors declare that they have no known competing financial interests or personal relationships that could have appeared to influence the work reported in this paper.
