# Supplementary material for: The value of PROMs for predicting erectile dysfunction in prostate cancer patients with Bayesian network
Source: Tech Innov Patient Support Radiat Oncol. 2024 Feb 13;31:100234. doi: 10.1016/j.tipsro.2024.100234 (PMC11345401; doi:10.1016/j.tipsro.2024.100234)
Supplement: Supplementary data 1 [file mmc1.docx]

# Supplementary Materials


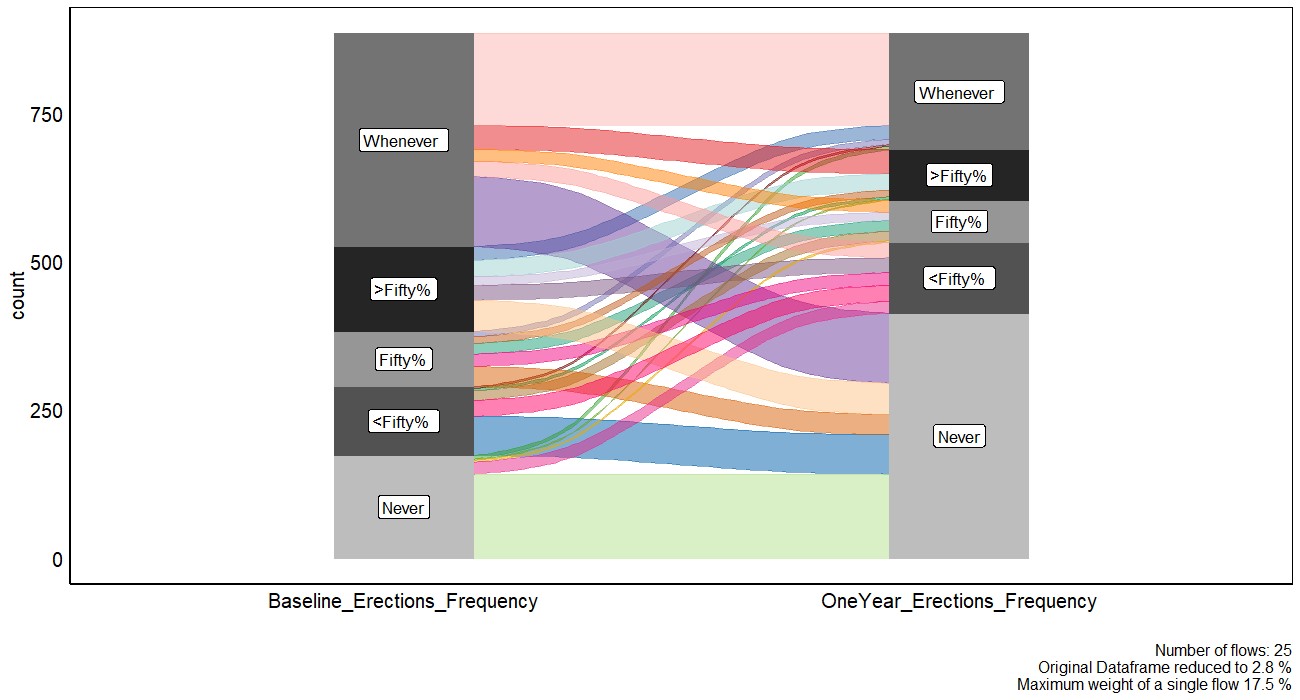

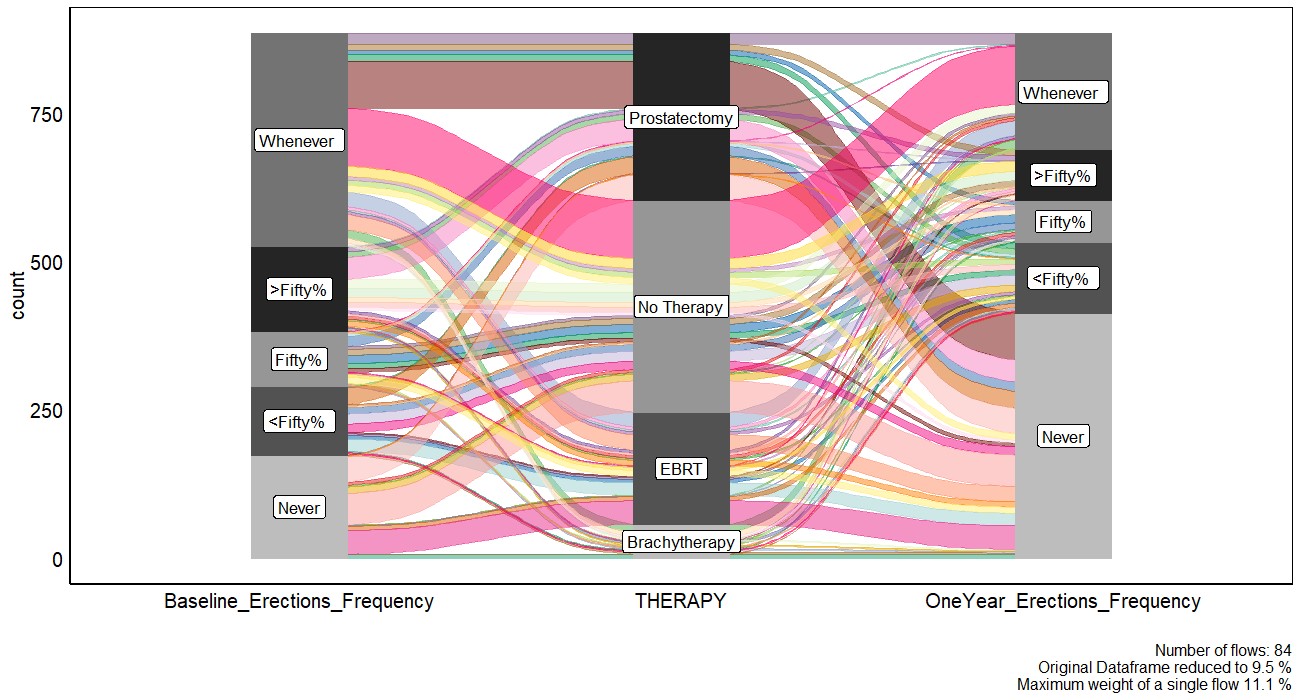


Figure S1: *Frequency of erections flow change in patients at baseline and 1-year.*


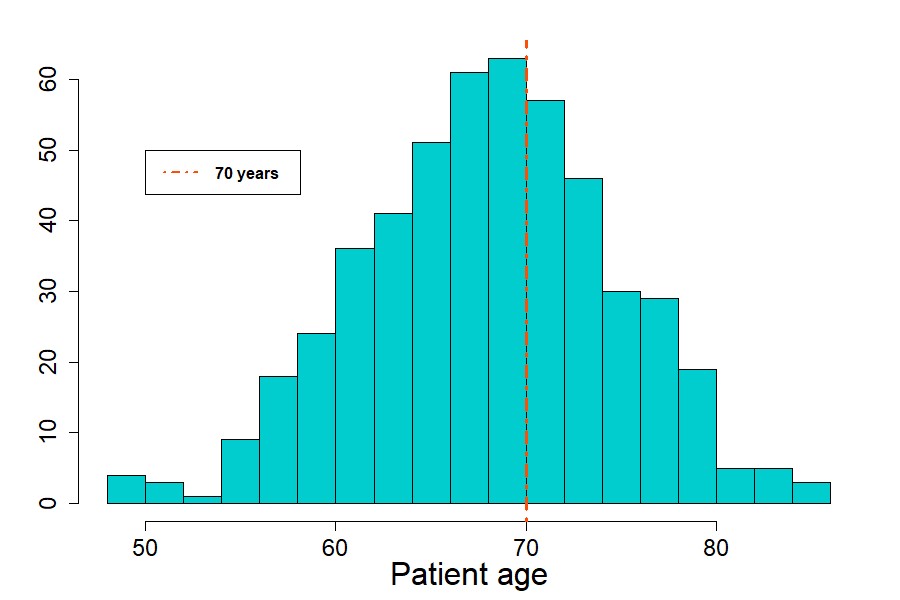

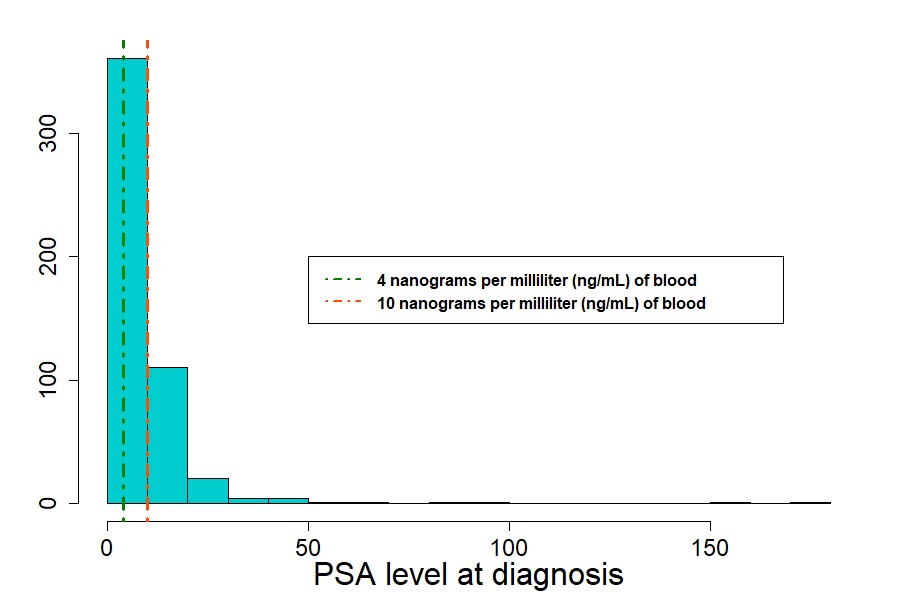


Figure S1: *Histogram of age and prostate-specific antigen level with cutoff thresholds .*


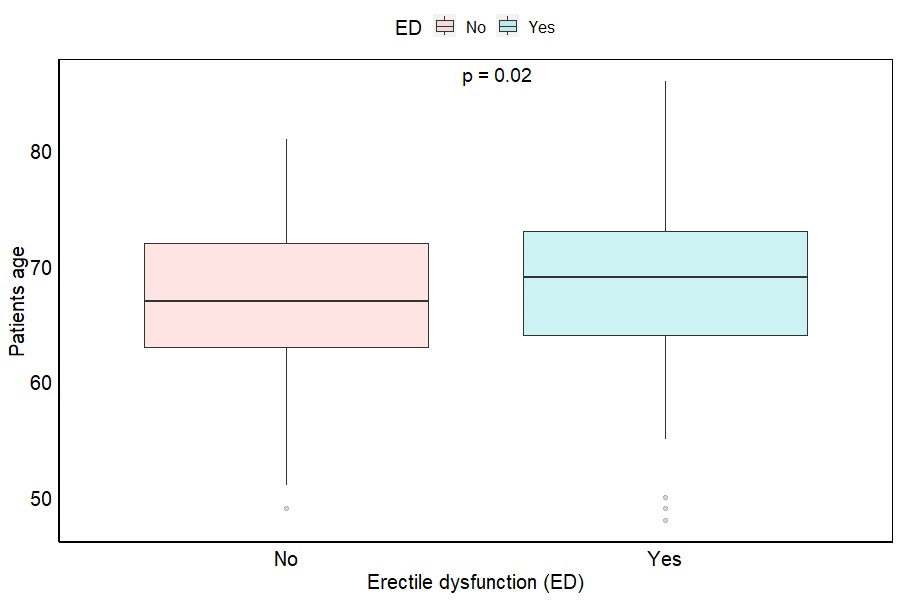

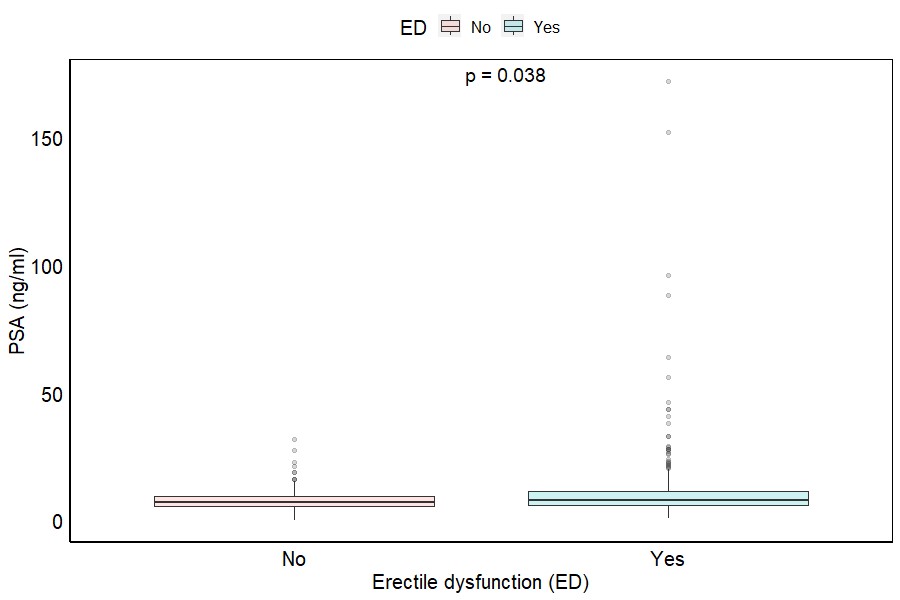


Figure S2: *Boxplot of age and prostate-specific antigen level by erectile dysfunction status.*


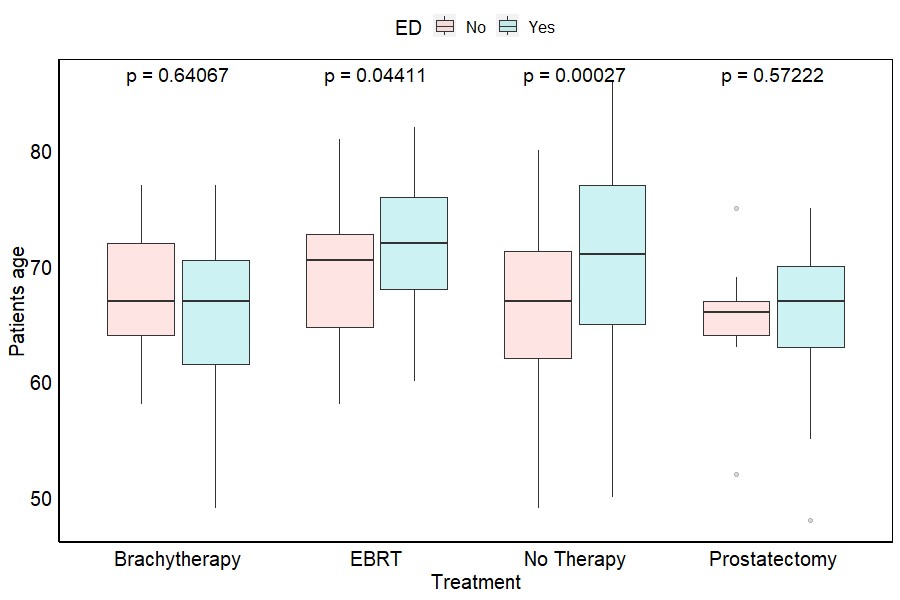

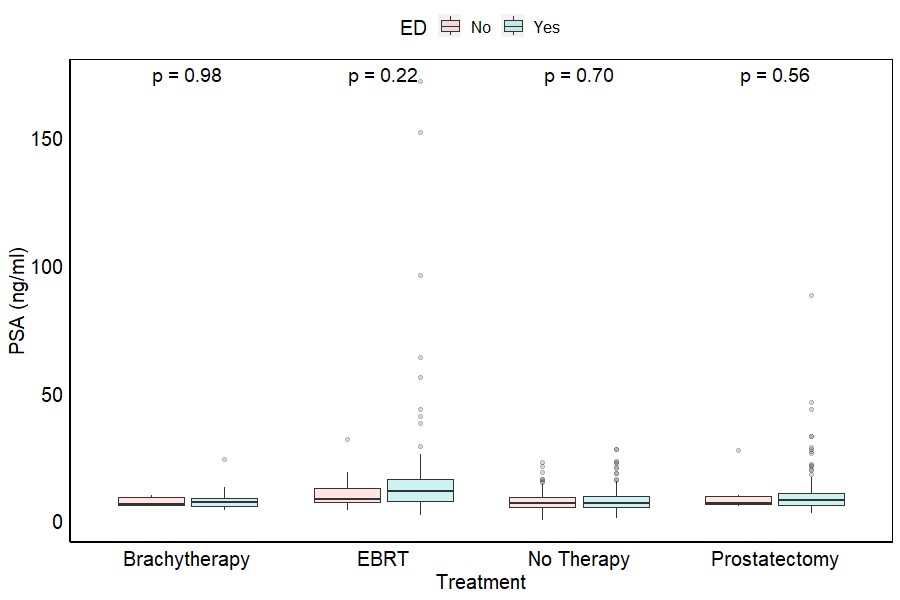


Figure S3: *Boxplot of age and prostate-specific antigen level by erectile dysfunction status for each treatment.*


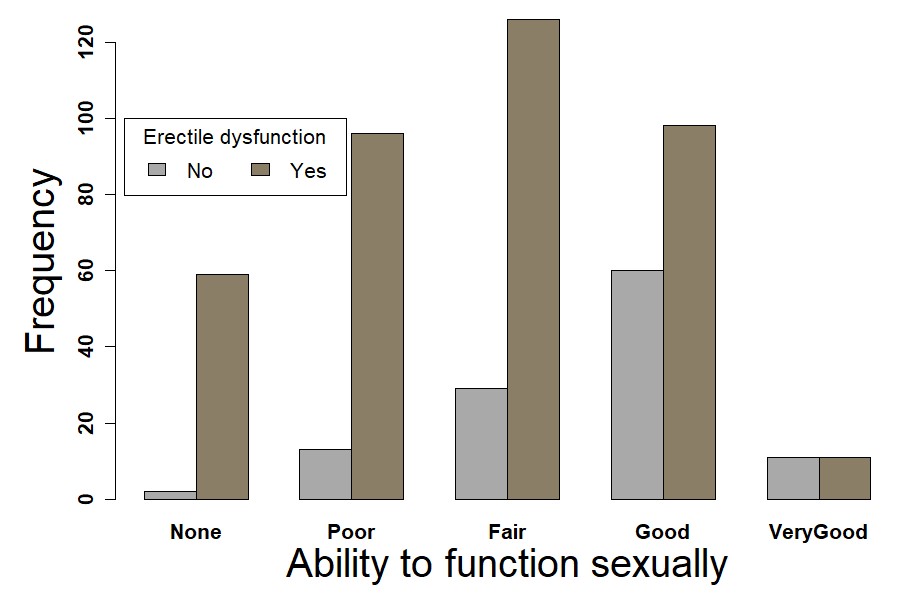

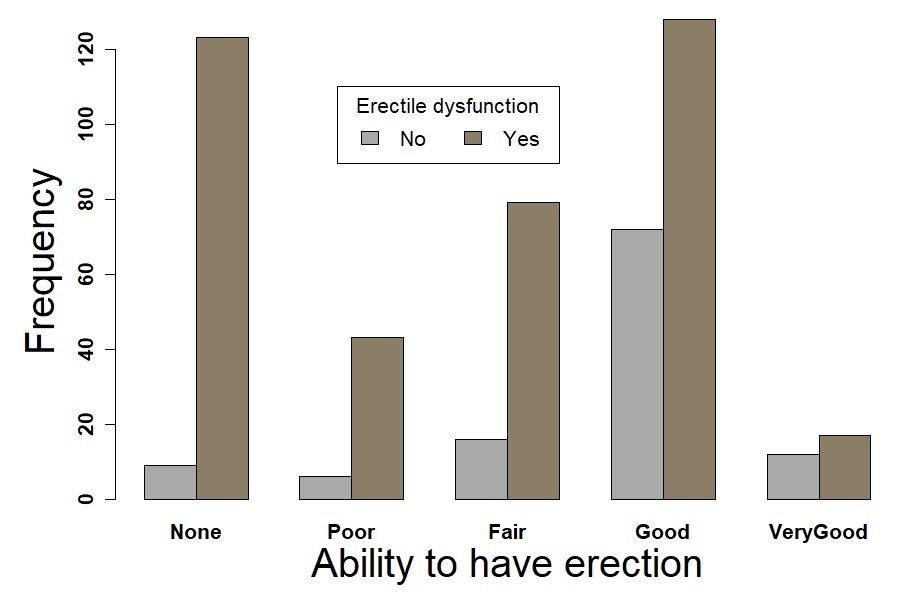

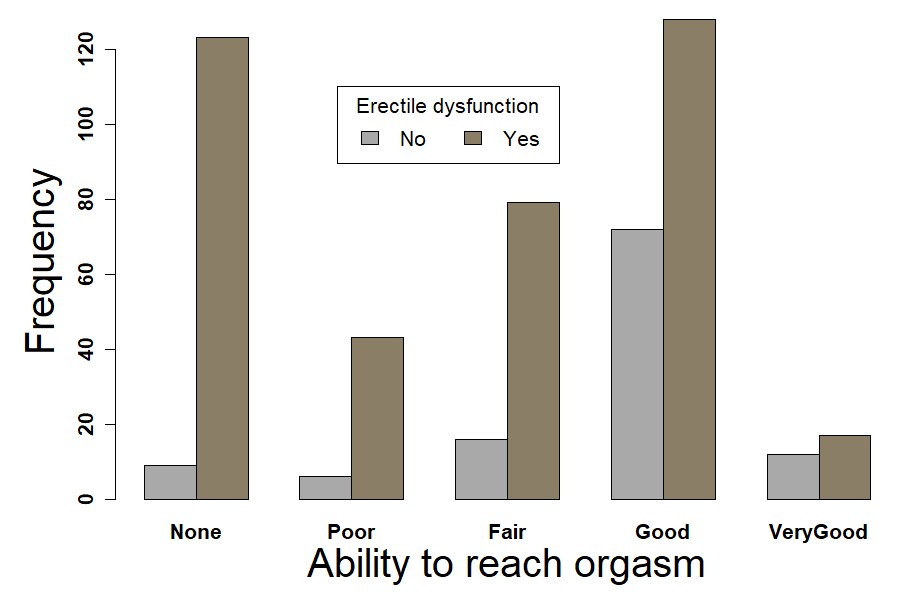

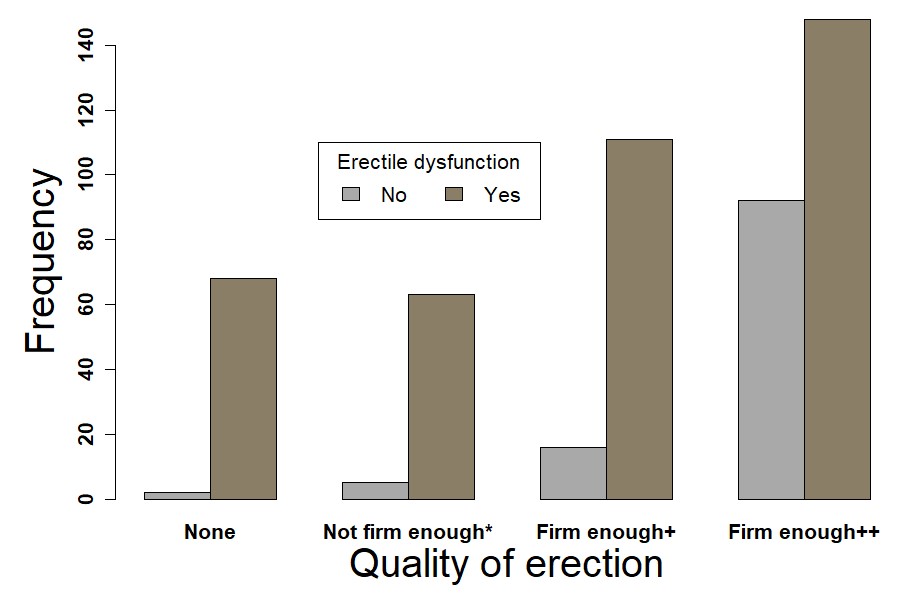

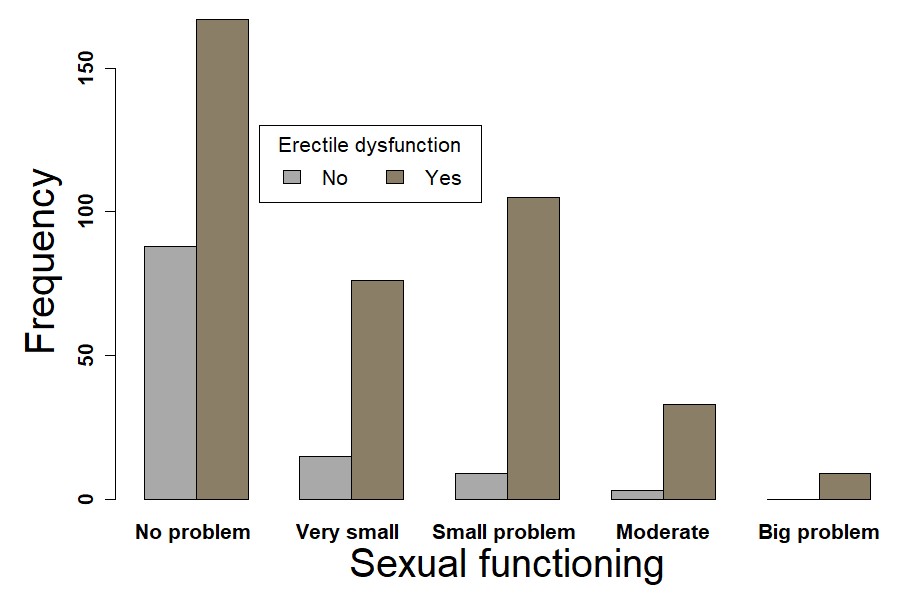

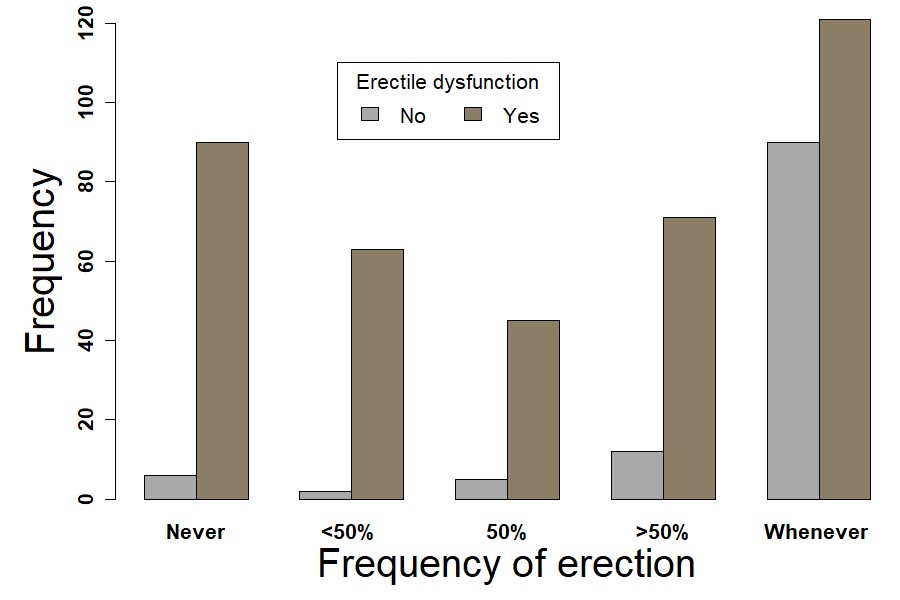

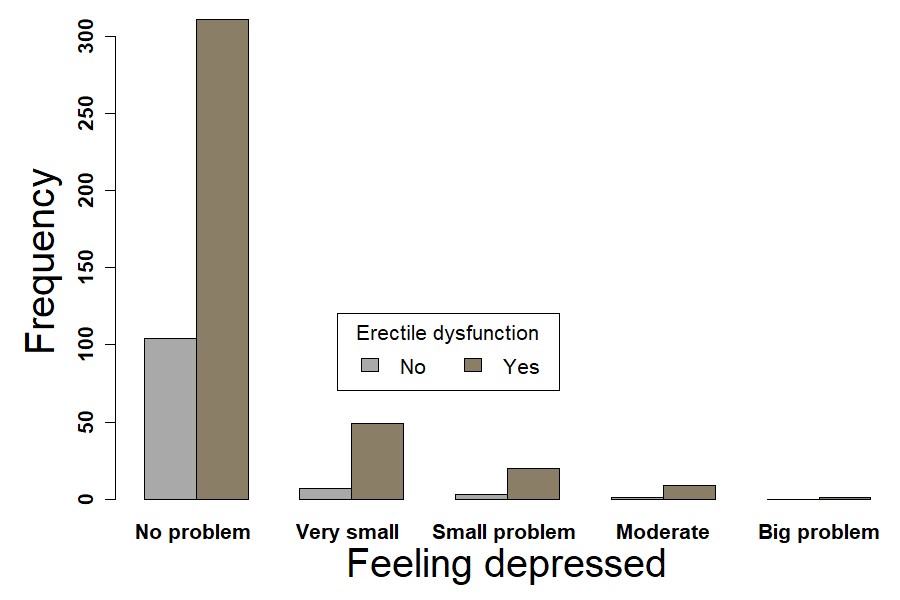


Figure S4: *Bar-plot of the considered PROM variables at baseline by erectile dysfunction status at 1-year.*


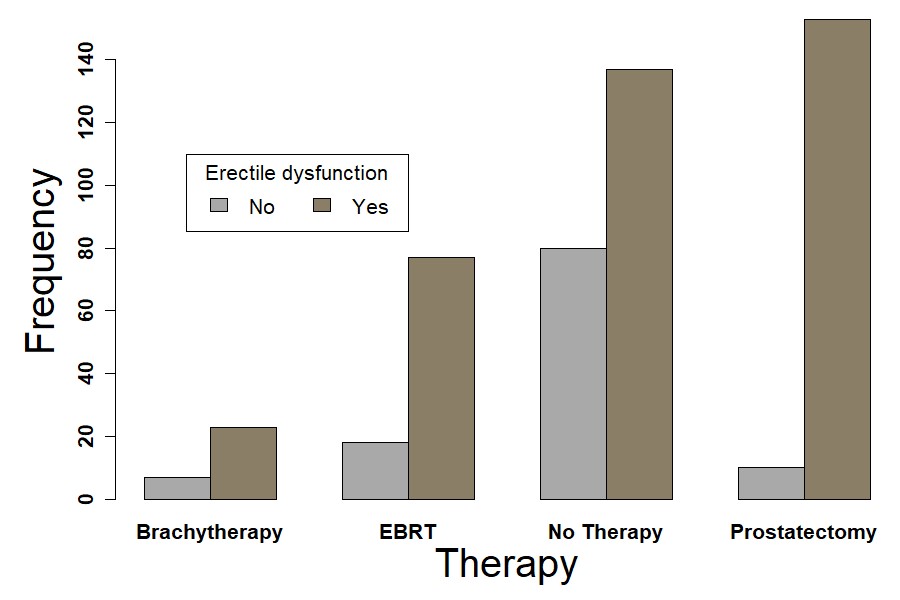

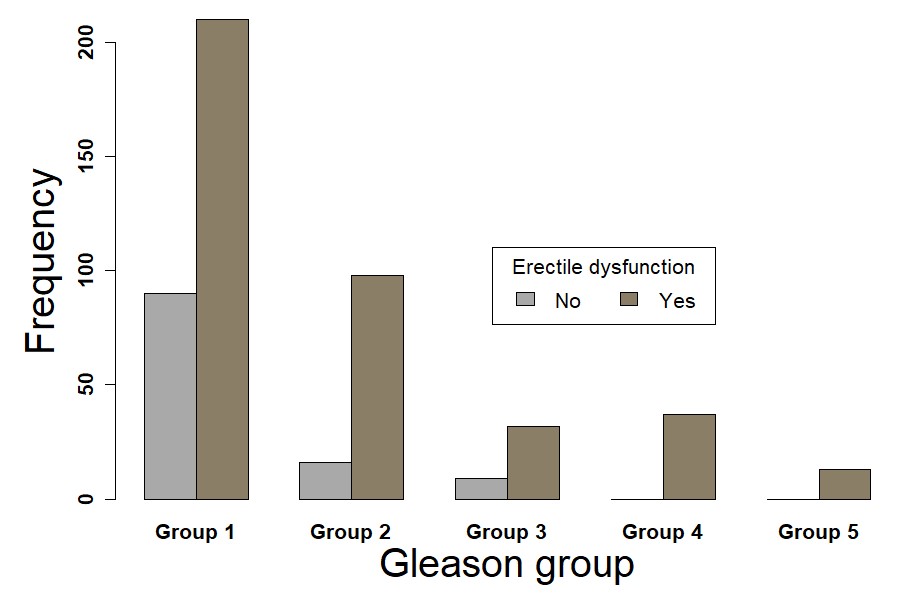

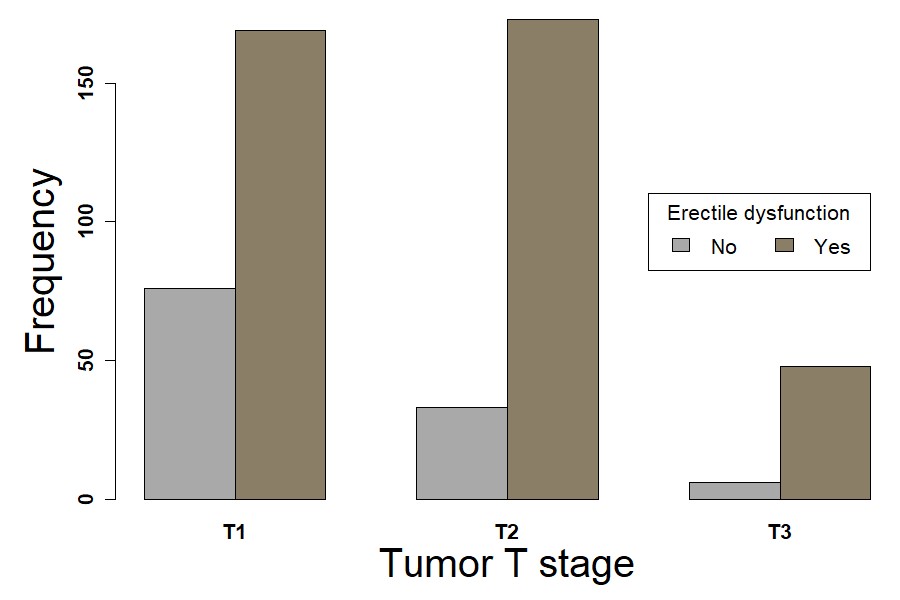

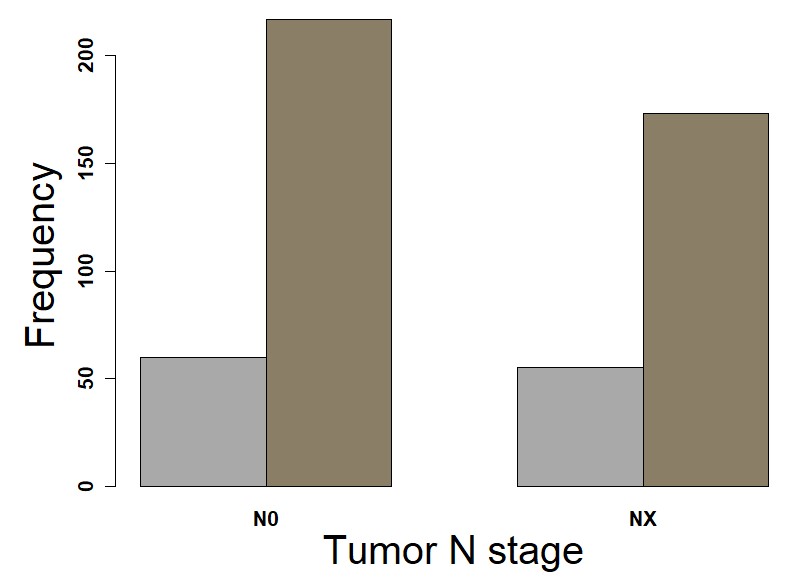

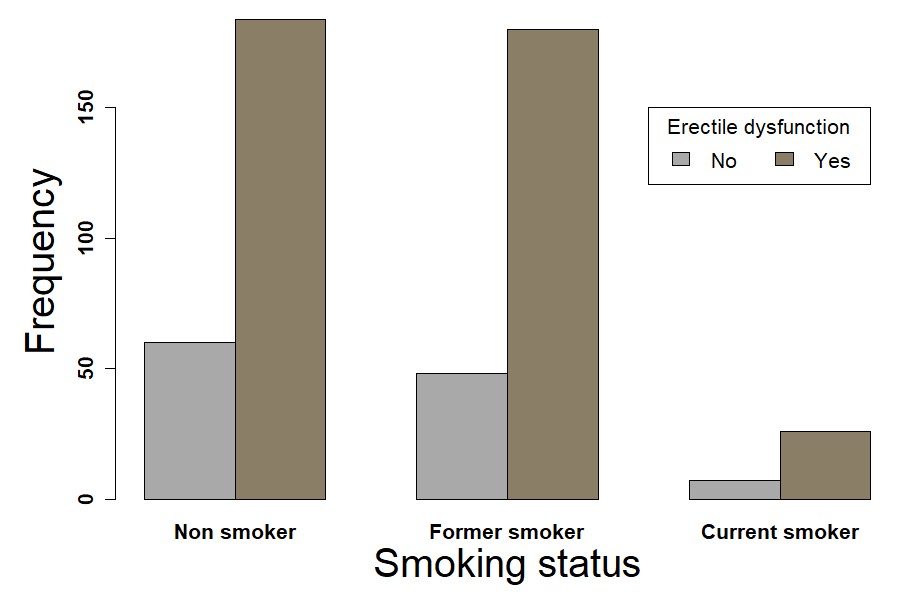

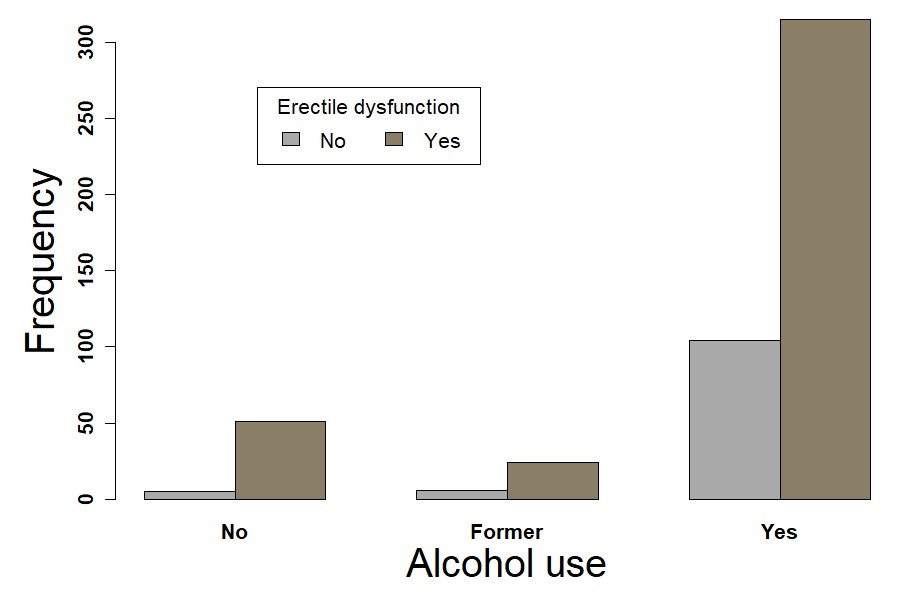

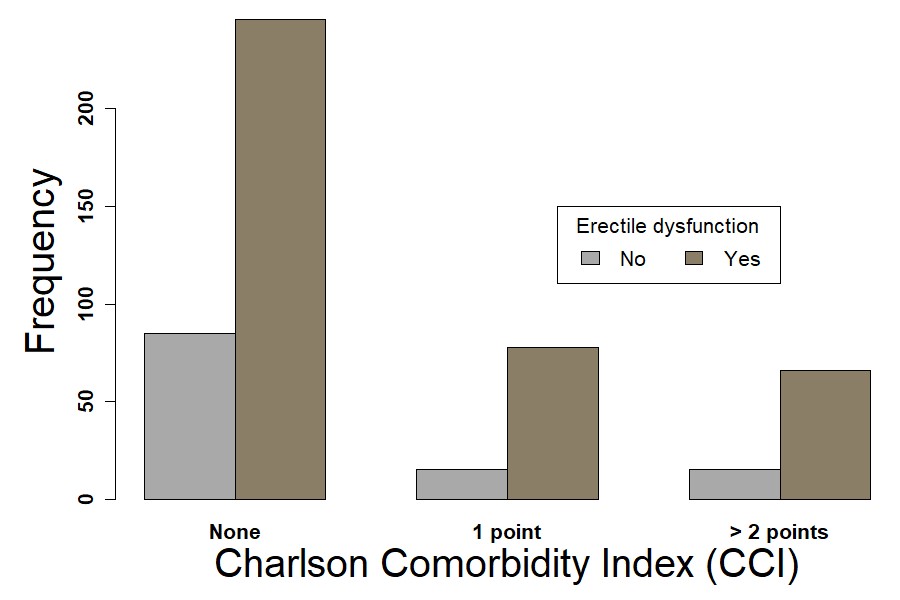

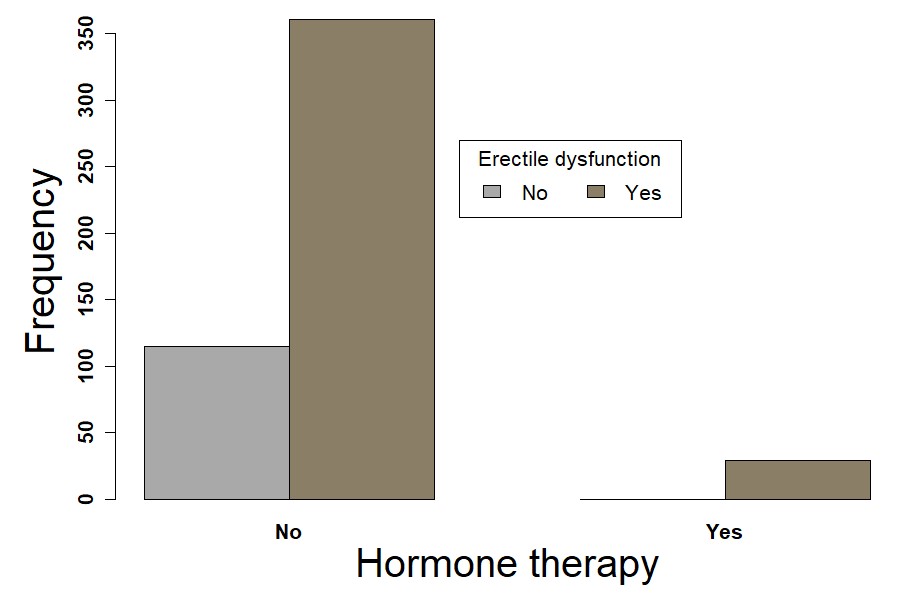

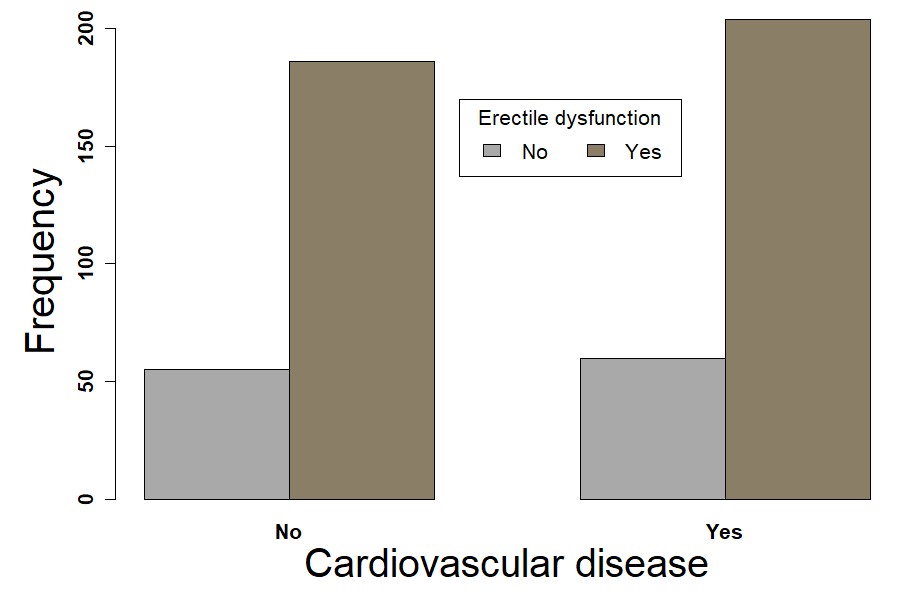

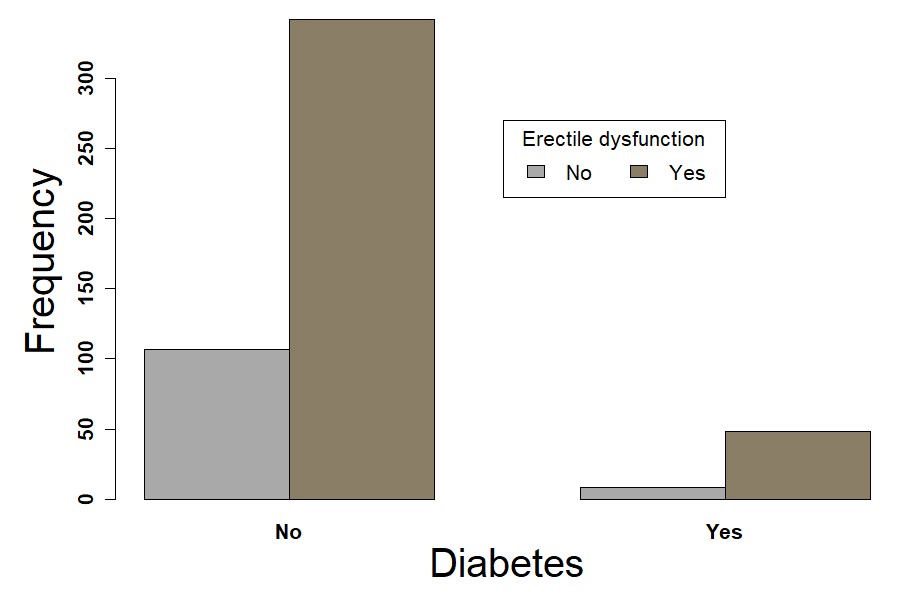


Figure S5: *Bar-plot of the considered clinical variables at baseline by erectile dysfunction status at 1-year.*


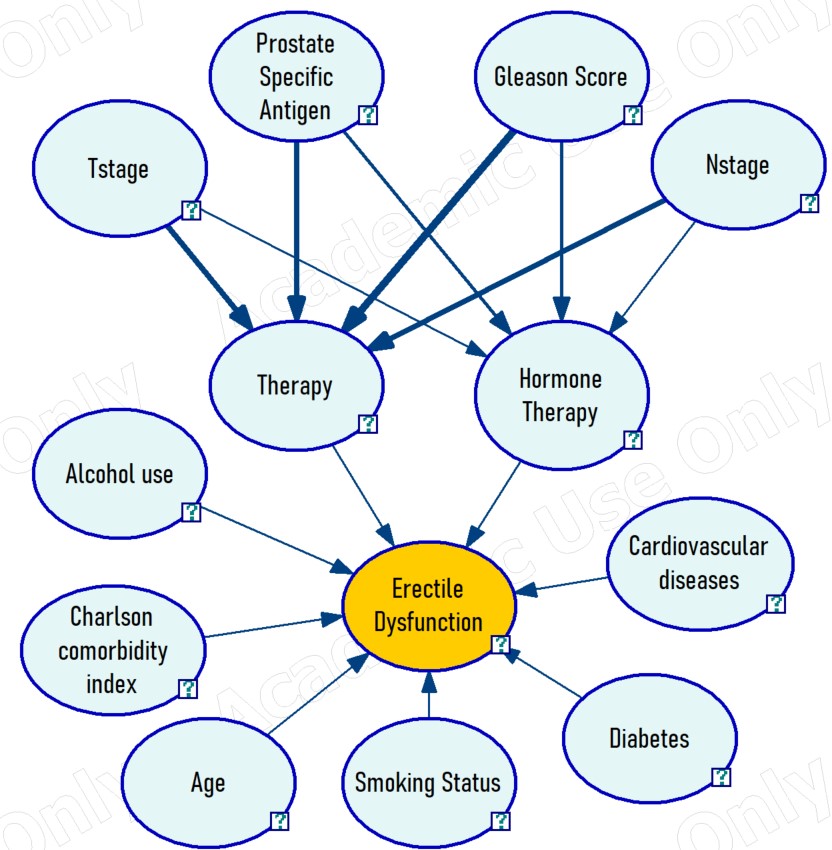

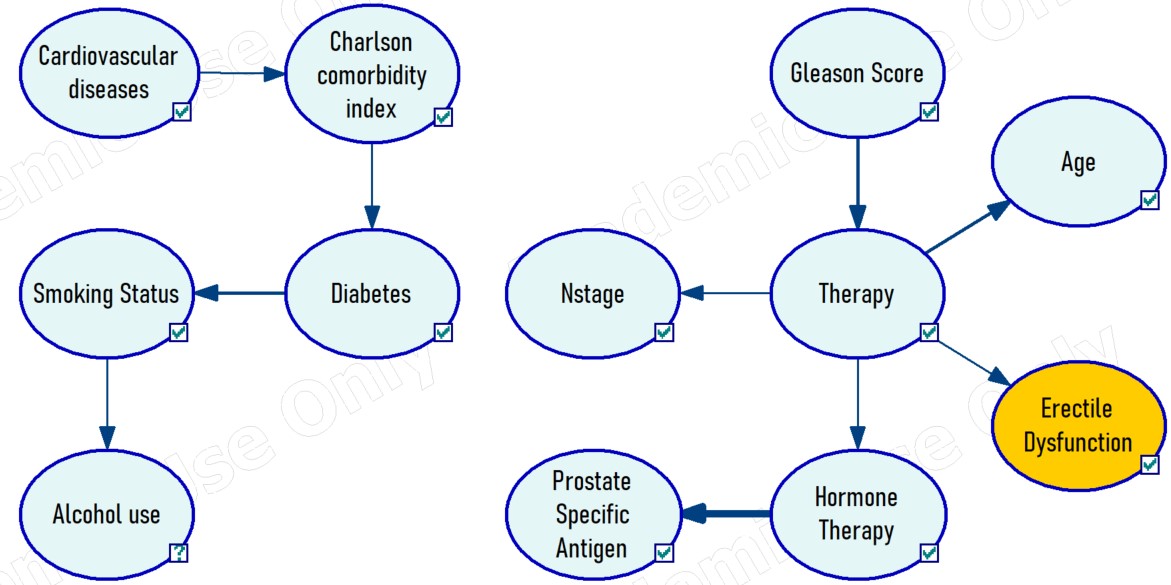


Figure S6: *Experts and algorithmic structure based on the clinical information. The arrow size indicates the magnitude of influence.*


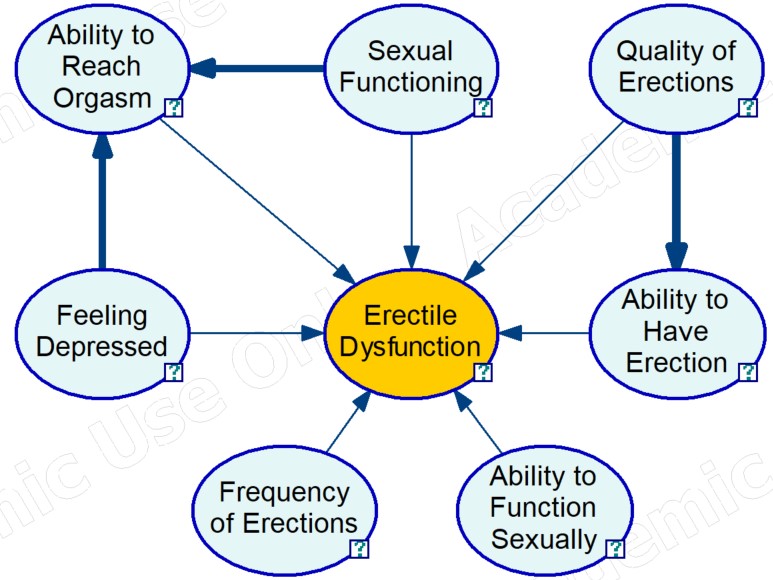

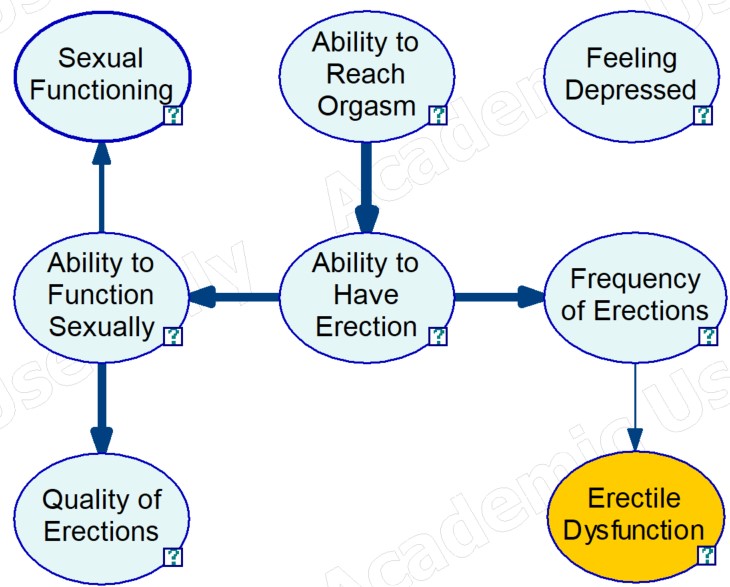


Figure S7: *Experts and algorithmic structure based on the PROM information. The arrow size indicates the magnitude of influence.*


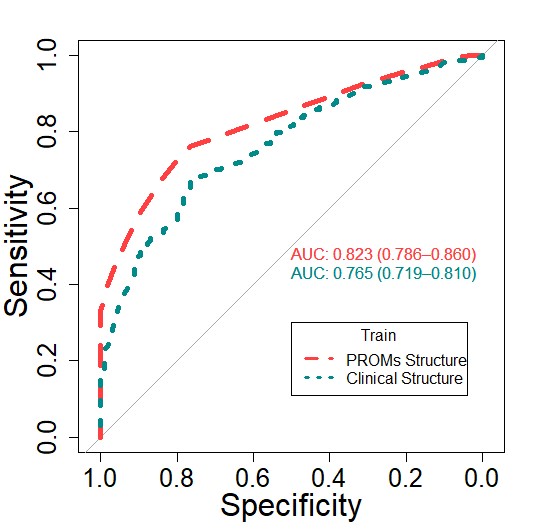

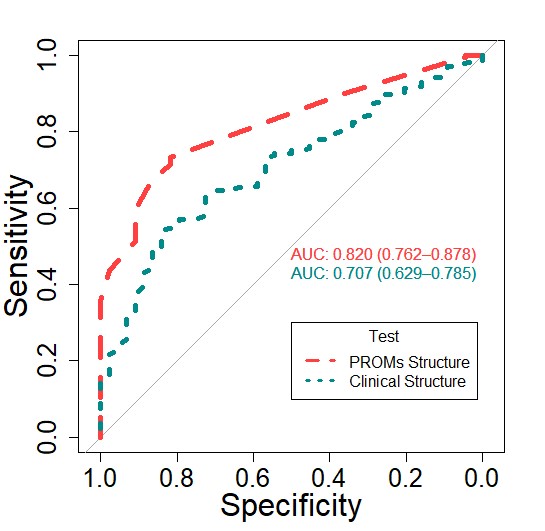


Figure S8: *AUC plots of the expert-modified structures on the train and test data*
